# Effect of depression disorder on the efficacy and quality of life of first-line chemotherapy combined with immunotherapy in oncogene-driver negative NSCLC patients

**DOI:** 10.3389/fonc.2022.772102

**Published:** 2022-07-25

**Authors:** Wen Li, Ziran Bi, Junxu Wu, Xu Duan, Lulian Pang, Yanyan Jing, Xiangxiang Yin, Huaidong Cheng

**Affiliations:** ^1^ Cancer Treatment Center, The Second Affiliated Hospital of Anhui Medical University, Hefei, China; ^2^ Department of Cardiothoracic Surgery, The Second Affiliated Hospital of Anhui Medical University, Hefei, China

**Keywords:** depression disorder, immunotherapy, chemotherapy, NCCLC, quality of life, first-line

## Abstract

**Objective:**

The current research was to assess the relevance between depression disorder and first-line chemotherapy combined with immunotherapy, quality of life in patients with oncogene-driver negative non-small cell cancer (NSCLC).

**Methods:**

NSCLC patients (33 with depression disorder and 34 with no depression disorder) who was received first-line chemotherapy combined with immunotherapy performed Zung Self-rating Depression Scale (SDS) and European Organisation for Research and Treatment of Cancer Quality of Life Questionnaire (EORTC QLQ-C30).

**Results:**

The Progression-Free Survival (PFS) of depression disorder group survivors were lower than these of no depression disorder group survivors (HR, 0.352; 95% CI, 0.201-0.617; P<0.05). The statistical significant was revealed about the Objective Response Rate (ORR) and Disease Control Rate (DCR) in two groups (P<0.05). The quality of life scores of NSCLC patients in no depression disorder group was significantly higher after chemotherapy combined with immunotherapy, and manifested as 92.7 ± 28 vs. 76.3 ± 23.3 (t=8.317, *P*<0.05), and had a significant difference.

**Conclusion:**

Depression disorder in oncogene-driver negative NSCLC patients influence the curative effect of chemotherapy combined with immunotherapy, and depression disorder was significantly negatively associated with quality of life following chemotherapy combined with immunotherapy.

## 1 Introduction

Non-small cell lung cancer(NSCLC) accounts for about 85% among all types of lung cancer, which was a disease with high morbidity and mortality (1). NSCLC was known for its high rates of depression disorder as well as a high degree of related physical symptomatology (2). Depression disorder was a serious mental illness, which including low mood, loss of interest, memory changes, low sense of self-worth, sleep disturbances and suicidal thoughts (3). It is suggested that about 16-29% of lung cancer patients experience depression disorder following treatment, and depressive was aggravated by tumor pain and disease progression (4). Empirical evidence found that depression disorder has become one of the important factors affecting the quality of life of lung cancer patients (5).

Serious researches had been confirmed that first-line chemotherapy in combination with programmed cell death protein 1(PD-1)/Programmed cell death protein 1 ligand (PD-L1) was the main method used in the therapy for patients with NCSCLC oncogene-driver negative (6). Gandhi et al (7) reported advances in immunotherapy research that pembrolizumab combined with chemotherapy significantly improved the overall survival, compared with chemotherapy alone for first-line treatment of oncogene-driver negative NSCLC patients, which further confirmed the safety and effectiveness for immunotherapy. PD-1/PD-L1 plays an important role in antitumor activity the modulation of the immune response in lung cancer cells, by blocking the PD-1/PD-L1 signaling pathway, the goal of cancer treatment was achieved (8, 9). The advantage of immunotherapy was the long duration of response, and once the treatment was effective, it can be maintained for years, even if the antitumor therapy remains stable (10). There was researches finding that patients with NSCLC showed cognitive and emotional deficits after chemotherapy, at the same time, brain network structure of frontal temporal lobe was changed (11). Currently, more and more attention has been paid to the depression disorder of NSCLC patients with negative oncogene-driver. Previous studies had found that 28.9% of lung cancer patients suffer from clinical depression, and depression disorder has a significant negative impact on the quality of life, and the quality of life in lung cancer survivors with depression can be improved through drug or psychological treatment (5). However, the relationship between depression disorder and quality of life in NSCLC patients with negative oncogene-driver following chemotherapy combined with immunotherapy were unknown.

In this study, we attempt to survey depression disorder and quality of life in 67 NSCLC patients with negative oncogene-driver who received chemotherapy combined with immunotherapy, and expound whether the depression disorder affected quality of life in negative oncogene-driver NSCLC survivors following chemotherapy combined with immunotherapy.

## 2 Methods

### 2.1 Participants

All 67 oncogene-driver negative NSCLC patients were admitted to the Second Affiliated Hospital of Anhui Medical University. According to the Zung self-rating depression scale (SDS)score (12), the oncogene-driver negative NSCLC patients were divided into two groups without (SDS=<39) and with (SDS > =40) depression disorder, and the groups were matched for age, education level and other factors. The Research Ethics Committee of the Affiliated Second Hospital approved the study (Number of Ethical Approval: 2 012088), and all subjects provided their informed consent.

The inclusion criteria for this study were as follows: 1. Patients with pathologically confirmed NSCLC who had not previously received chemotherapy combined with immunotherapy; 2. The dose and time of chemotherapy combined with immunotherapy followed the standard, and the estimated survival time was more than half a year; 3. Patients were 18 years old at diagnosis; 4. Karnofsky performance status (KPS) score ≥70, which was able to communicate normally without language barriers.

The exclusion criteria for this study were as follows: 1. Patients with diseases that influenced quality of life but were not related to the tumor itself, including fractures, cerebral infarction, cardiac insufficiency, etc. 2. anxiety, dementia and other mental illnesses and 3. Others ill with mental disorder, which seriously affecting the quality of life.

### 2.2 Procedure

Oncogene-driver negative NSCLC patients were identified by prescreening inpatient tumor data, and qualified patients were recruited during hospitalization. After the oncologist made an investigative presentation to the patient and obtained informed consent. Patients’ ability to participate was assessed, baseline data were collected, and questionnaires were issued. The SDS assess patients before starting their first treatment. European Organisation for Research and Treatment of Cancer Quality of Life Questionnaire (EORTC QLQ-C30) questionnaire was completed before the start of chemotherapy combined with immunotherapy and at the time of the first disease progression after treatment. Treatment outcomes were evaluated every two cycles by standard Response Evaluation Criteria in Solid Tumors (RECIST). The efficacy of chemotherapy combined with immunotherapy was evaluated for each 2 courses of treatment.

### 2.3 Measures

#### 2.3.1 Evaluation of depression disorder

To assess patients’ subjective perceptions of their depressive symptoms, the SDS depression scale was used (13). SDS consists of 20 items designed based on diagnostic criteria for depression. Subjects rated each item on a Likert 4-scale based on how they had felt in the past few days. The original sum of SDS ranges from 20 to 80, and 40 is the cut point according to research. Those with a score less than 40 were not depressed, and those with a score greater than or equal to 40 were depressed, and the higher the score was, the more severe the depression was.

#### 2.3.2 Quality of life evaluation

EORTC QLQ-C30 is a cross-cultural and cross-national core scale for assessing cancer patients’ quality of life (14). There are 30 items in total (1-4 points for each item), which are divided into 15 areas (five functional areas, three symptom areas, one overall quality of life, six single item areas). Each field is converted into a standard score of 0~100 points for observation. Higher scores in functional areas were associated with better quality of life, while higher scores in symptom areas were related with poorer quality of life (15). The specific calculation method is as follows: First, the raw score (RS) is calculated according to each sub-scale, RS= (I1+I2+I3+…+In)/n. Then, the score of 0-100 was linearly transformed, and the score of each subscale was calculated as follows: Functional subscale S={1-(RS-1)/range}*100; Symptom subscale: S={(RS-1)/range}*100; Overall quality of life subscale: S={(RS-1)/range}*100. Where range represents a very poor score, with a very poor score of 3 for the functional and symptom subscales and a very poor score of 6 for the overall quality of life subscale. The total score of EORTC QLQ-C30 is calculated as the average of the total scores of 13 scales (excluding the economic difficulties in a single item and the overall quality of life dimension). The higher the score is, the better the quality of life (16). The fine reliability and validity of EORTC QLQ-C30 in the evaluation of quality of life are confirmed for cancer patients (17).

#### 2.3.3 Evaluation of the efficacy of immunotherapy

Efficacy was evaluated by RECIST criteria. The criteria divided the evaluation of target lesions into four levels, including complete response (CR), partial response (PR), stable disease (SD) and progressive disease (PD) (18).

### 2.4 Statistical analysis

Statistical analysis SPSS statistical software was used for statistical analysis. The results of the analysis were expressed as mean ± standard deviation in the study. The scores in the depression disorder group and the no depression disorder group were compared using two independent samples t tests. The correlation between covariates and survival was evaluated by Cox regression analysis using deleted data.

## 3 Results

### 3.1 Baseline demographics and clinical data

**Figure 1:**
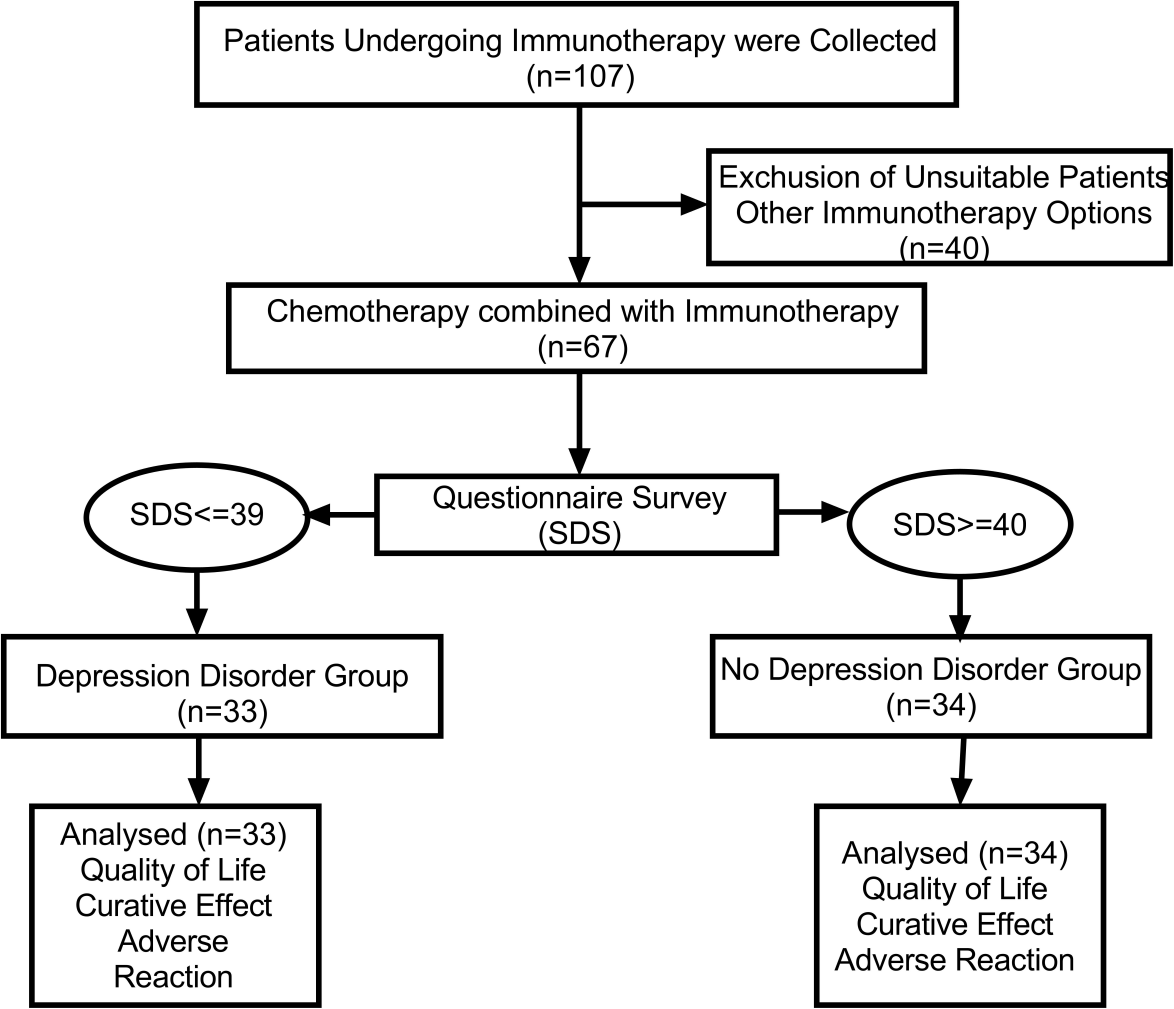
Research Flowchart. SDS, Self-Rating Depression Scale.

According to **Figure 1**, The study included 107 patients, 67 of whom were included in the combined immunochemotherapy regimen, including 33 in the depression disorder group and 34 in the non-depression disorder group. According to **Table 1**, In age between the two groups (t = 1.492, P = 0.140), sex (χ2 = 0.165, P = 0.105), education (χ2 = 3.521, P = 0.318), KPS (χ2 = 0.729, P = 0.393), pathological type (χ2 = 1.214, P=0.271), tumor stage (χ2 = 0.441, P=0.507) and other demographic information were not statistically significant.

**Table 1:** The clinical data of NSCLC patients in the depression disorder group and the no depression disorder group.

Characteristic	No DD (n=34)	DD (n=33)	T/χ2	P
Age	62.15 ± 9.54	65.67 ± 9.77	-1.492	.140
Sex, n (%)			0.165	.648
male	30(88)	28(85)		
female	4(12)	5(15)		
Education, n (%)			3.521	.318
illiteracy	4(12)	8(24)		
primary school	14(41)	12(36)		
middle school	10(29)	11(33)		
university	6(18)	2(7)		
Pathology, n (%)			1.214	.271
adenocarcinoma	15(44)	19(58)		
squamous cell carcinoma	19(56)	14(42)		
others	0(0)	0(0)		
Tumor stage, n (%)			0.441	.507
III	6(18)	8(24)		
IV	28(82)	25(76)		
KPS, n (%)			0.729	.393
80	15(44)	18(55)		
90	19(56)	15(45)		

DD, depression disorder group.

### 3.2 Comparison of therapeutic effect between the two groups

**Figure 2:**
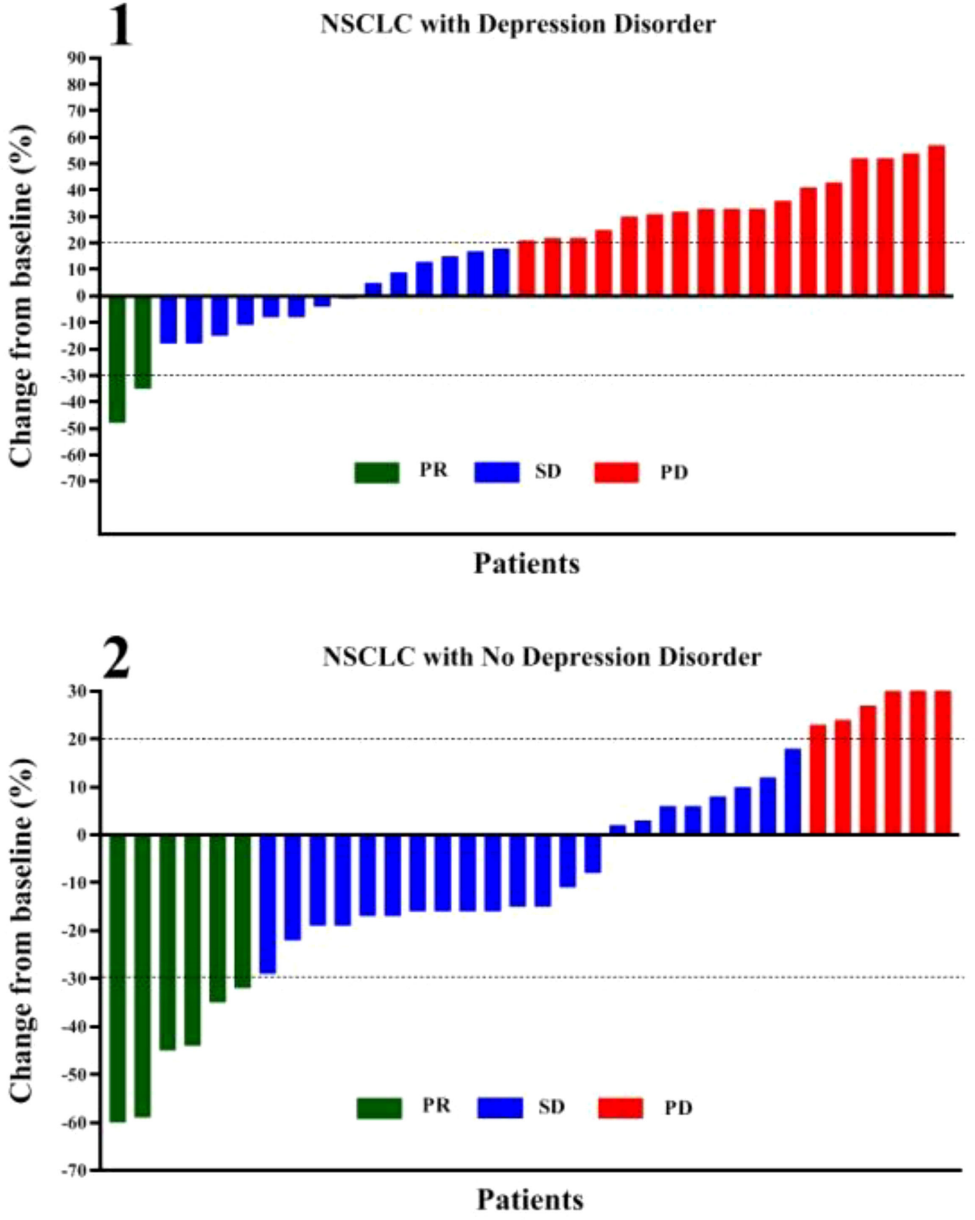
Correlation between depression disorder and chemotherapy combined with immunotherapy in patients with oncogene-driver negative NSCLC. PR, partial response; SD, stable disease; PD, progressive disease.

According to **Figure 2** and **Table 2**, in the depression disorder group, 2 cases achieved PR, 14 cases achieved SD, and 17 cases achieved PD. In the group without depression disorder, 6 achieved PR, 22 achieved SD and 6 achieved PD. The ORR and DCR of the non-depressive disorder group were 17.6% and 82.4%, while the ORR and DCR of the depressive disorder group were 6.1% and 48.5%, the difference was statistically significant (P<0.05)).

**Table 2:** The efficacy of chemotherapy combined with immunotherapy in oncogene-driver negative NSCLC patients in the depression disorder group and no depression disorder group.

Efficacy Outcome	Group	
	Depression Disorder (n=33)	No Depression Disorder (n=34)
PR	2(6%)	6(18%)*
SD	14(42%)	22(64%)
PD	17(52%)	6(18%)
ORR	6.1%	17.6%
DCR	48.5%	82.4%

*P<0.05.CR, complete response; PR, partial response; SD, stable disease; PD, progressive disease; ORR, objective response rate; DCR, disease control rate.

### 3.3 The correlation between depression disorder and quality of life

**Figure 3:**
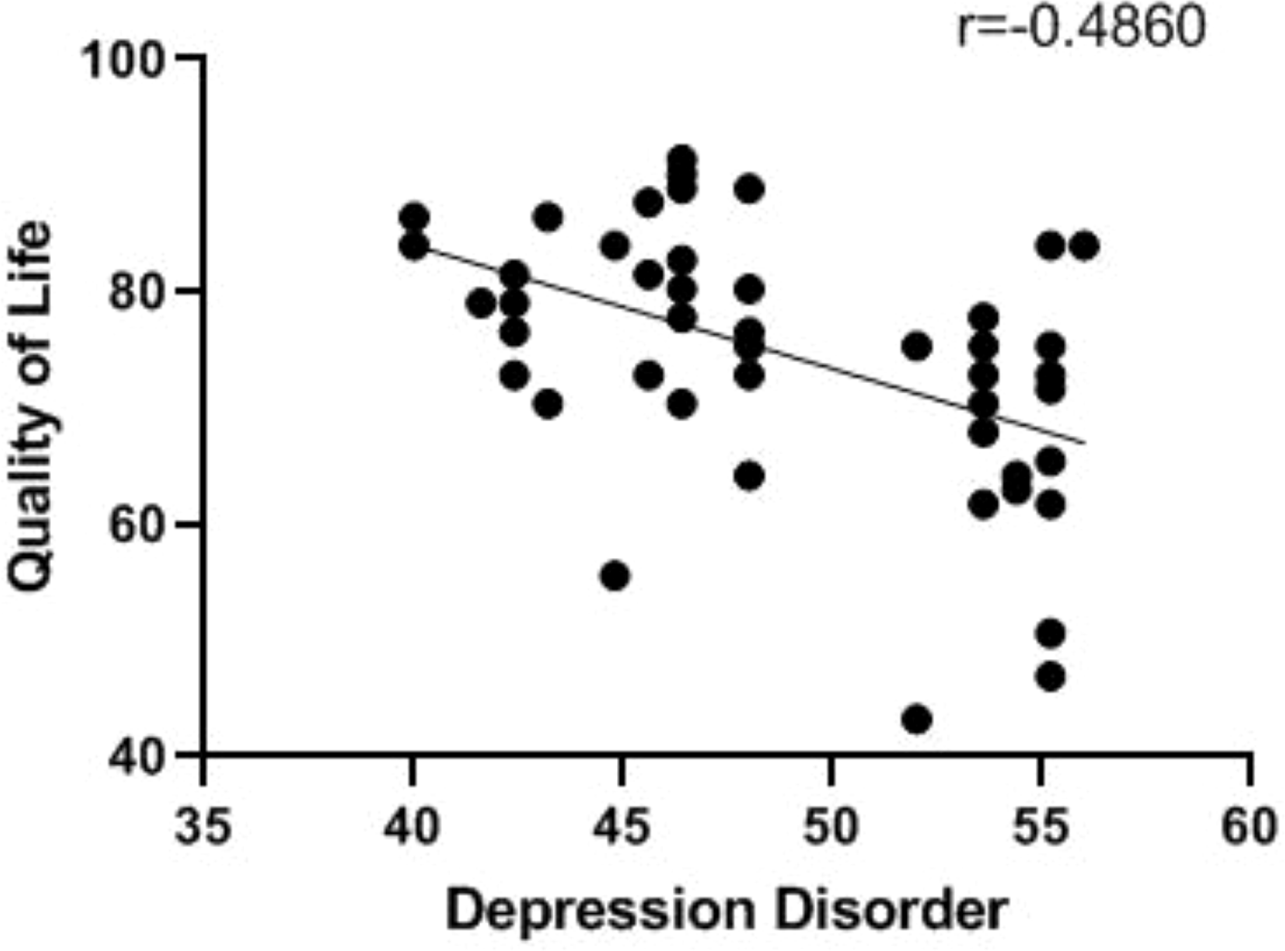
Relationship between depression disorder and quality of life after chemotherapy combined with immunotherapy.

We can see this in **Table 3**, the quality of life scores of NSCLC patients in no depression disorder group was significantly higher before chemotherapy combined with immunotherapy, and manifested as 91.0 ± 28.3 vs. 81.7 ± 23.7 (t=4.985, P<0.05), and had a significant difference. The quality of life scores of NSCLC patients in no depression disorder group was significantly higher after chemotherapy combined with immunotherapy, and manifested as 92.7 ± 28 vs. 76.3 ± 23.3 (t=8.317, P<0.05), and had a significant difference. **Figure 3** shows that there was a negative correlation between the depression disorder score and the quality of life score in patients after chemotherapy combined with immunotherapy (r=-0.4860, P <0.001).

**Table 3:** Comparison of quality of life between patients with and without depressive disorder before and after treatment.

N	DD	No DD	t	P
	33	34		
EORTC QLQ-C30 summary score (per 10 points) 1	81.7 ± 23.7	91.0 ± 28.3	4.985	<0.001
EORTC QLQ-C30 summary score (per 10 points) 2	76.3 ± 23.3	92.7 ± 28	8.317	.<0.001

DD, depression disorder group; EORTC QLQ-C30 summary score (per 10 points) 1: before chemotherapy combined with immunotherapy; EORTC QLQ-C30 summary score (per 10 points) 2: after chemotherapy combined with immunotherapy.

### 3.4 The correlation between depression disorder and adverse events

As shown in **Table 4**, adverse events were similar in the two groups of patients with oncogene-driver negative NSCLC: the proportion of patients with hematologic syndromes in the depression disorder group was 9% and that in the group without depression disorder group was 12%. Meanwhile, the incidences of immune-related dermatitis (33% vs. 9%), immune-related pneumonitis (6% vs. 0) and immune-related enteritis (6% vs. 0) were significantly higher in the depression disorder group than in the group without depression disorder.

**Table 4:** Effects of depression disorder on adverse reactions during chemotherapy combined with immunotherapy in NSCLC patients with oncogene-driver negative.

Adverse event	NO.%		P
	DD (n=33)	No DD (n=34)	
Hematologic syndromes	3(9)	4(12)	.721
Immune-related dermatitis	11(33)	3(9)	.014
Immune-related pneumonitis	2(6)	0(0)	.145
Immune-related enteritis	2(6)	0(0)	.145
Thyroid dysfunction	1(3)	0(0)	.306
Others	2(6)	0(0)	.145

DD, depression disorder group.

### 3.5 PFS in patients receiving treatment

**Figure 4:**
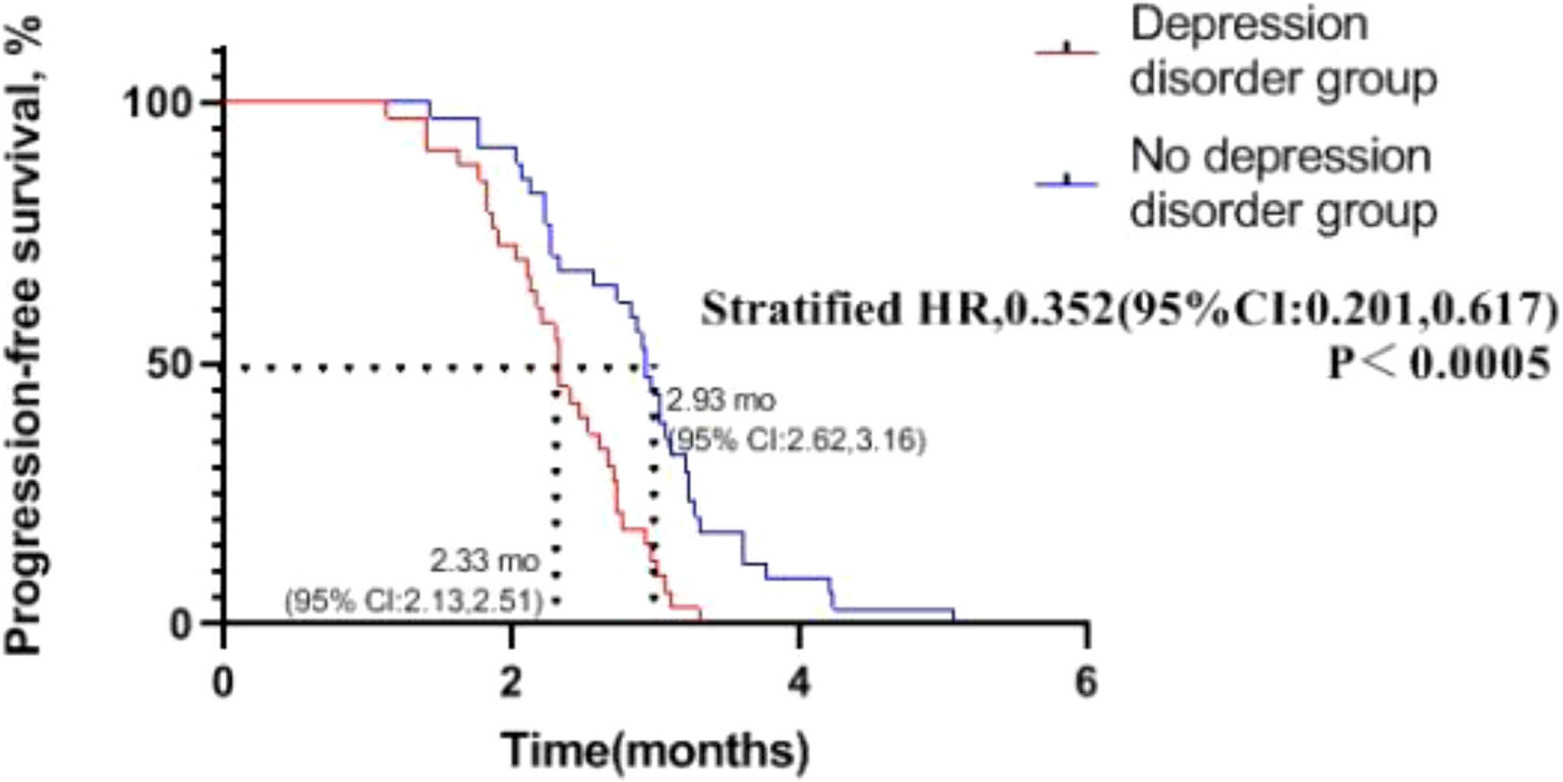
Progression-free survival in patients with oncogene-driver negative non-small cell lung cancer receiving chemotherapy combined with immunotherapy.

We can see from **Figure 4** that in patients with advanced NSCLC who received chemotherapy combined with immunotherapy, median of PFS of depression disorder group is 2.33 months, and median of PFS of non- depression disorder group is 2.93 months, PFS in the non- depression disorder group was significantly better than that in the depression disorder group (HR, 0.352; 95% CI, 0.201-0.617; P <0.05).

## 4 Discussion

This study suggests that depression disorder is one of the factors influencing the quality of life and prognosis of NSCLC patients receiving chemotherapy combined with immunotherapy with negative oncogene-driver. We found that the ORR, DCR and PFS of patients with depression disorder were significantly lower than those of patients without depression disorder. At the same time, we found that depression disorder was negatively associated with quality of life in NSCLC patients with negative oncogene-driver after chemotherapy combined with immunotherapy.

Depression disorder is defined as a multifactorial, unpleasant experience of a psychological (i.e., cognitive, behavioral, emotional), social, spiritual, and/or physical nature that may interfere with the ability to effectively cope with cancer, its physical symptoms, and treatment (19). Some studies suggest that monitoring for psychological distress should be as routine as monitoring for other vital signs (20).. Depression disorder is one of the important components of psychological distress. We usually use the SDS scale to monitor the level of depression disorder. According to the SDS scale score, we can know that many lung cancer patients have experienced depression disorder (21, 22).

Immunotherapy is an attempt to boost the immune system so that it can respond more effectively. Immunotherapy can be classified as active or passive in nature, depending on its interaction with the host immune system and the type of response it elicits. Active immune responses include humoral and/or cell-mediated immunity. In contrast, passive immune responses do not require activation of the immune system and stimulate the elimination of tumor cells by passively injecting a combination of pre-prepared antitumor immunoglobulin with tumor-associated antigens. For NSCLC patients with negative oncogene-driver, immunotherapy can be combined with chemotherapy (23), targeted therapy, and radiotherapy.

The efficacy of tumor chemotherapy combined with immunotherapy is affected by many factors. First, it is related to human immunity, which is closely related to genetics and the body’s microflora (24, 25). Second, it is related to tumor cells, intra-tumor heterogeneity of tumor neoantigen, amount of clonal neoantigen, mutation target of tumor cells, and mutation load of tumor significantly affect the therapeutic effect, among which patients with low intra-tumor heterogeneity of tumor neoantigen and high amount of clonal neoantigen have more therapeutic advantages (26). The third is related to environmental factors, such as daily living habits, eating habits, bacterial infection, drug dose type and so on. However, no research on the effect of depression disorder on chemotherapy combined with immunotherapy has been proposed.

Chemotherapy is a drug that directly poisons the DNA of cancer cells by taking advantage of the fact that cancer cells divide faster than normal healthy cells and expose their DNA. They have side effects by indiscriminately killing replicating cells (healthy or cancerous). Common side effects are fatigue, diarrhea, neuropathy and cytopenia. The common adverse reactions of immunotherapy include dermatitis, enteritis, endocrine disorders, pneumonia, hepatitis, fatigue and so on (27–33). In this study, we can see that the incidence of immune dermatitis in the depression disorder group is significantly higher than that in the non- depression disorder group, which may be related to depression disorder.

## 5 Study limitations

This study is the first to evaluate the effect of depression disorder on the efficacy and quality of life of chemotherapy combined with immunotherapy in NSCLC patients with negative oncogene-driver using SDS scale and EORTC QLQ-C30 scale. This study found a significant correlation between depression disorder and quality of life in NSCLC patients with negative oncogene-driver during chemotherapy combined with immunotherapy, but there are still some deficiencies. The study was a study of a small sample. Large sample sizes and longitudinal studies are needed to determine the exact relationship between depression disorder and efficacy of chemotherapy combined with immunotherapy and quality of life in patients with oncogene-driver negative NSCLC.

## 6 Conclusion

In short, our study provides direct evidence that depression disorder affects the quality of life of patients with oncogene-driver negative NSCLC during chemotherapy combined with immunotherapy, and provides a theoretical basis for improving the quality of life of oncogene-driver negative NSCLC survivors. At the same time, we found that depression disorder is one of the factors affecting the efficacy and adverse reactions of chemotherapy combined with immunotherapy in NSCLC patients with negative oncogene-driver, which provides a theoretical basis for improving the efficacy of chemotherapy combined with immunotherapy and reducing adverse reactions in patients with advanced NSCLC.

## Data availability statement

The original contributions presented in the study are included in the article/supplementary material. Further inquiries can be directed to the corresponding author.

## Ethics statement

The study was approved by the research ethics committee of the Affiliated Second Hospital of Anhui Medical University (Number of Ethical Approval: 2012088). The patients/participants provided their written informed consent to participate in this study.

## Author contributions

WL and ZB collected data, wrote manuscripts and answer reviewers questions; JW provided the data of the subjects and analyzed the results; XD and LP performed the basic information of participants collection; YJ and XY performed literature collection. HC designed the project. All authors contributed to manuscript editing.

## Funding

This research was supported by the National Natural Science Foundation of China (No. 81872504).

## Conflict of interest

The authors declare that the research was conducted in the absence of any commercial or financial relationships that could be construed as a potential conflict of interest.

## Publisher’s note

All claims expressed in this article are solely those of the authors and do not necessarily represent those of their affiliated organizations, or those of the publisher, the editors and the reviewers. Any product that may be evaluated in this article, or claim that may be made by its manufacturer, is not guaranteed or endorsed by the publisher.
